# Honing in on enteric fever

**DOI:** 10.7554/eLife.03545

**Published:** 2014-07-01

**Authors:** Lyle R McKinnon, Quarraisha Abdool Karim

**Affiliations:** 1 **Lyle R McKinnon** is in the Centre for the AIDS Programme of Research in South Africa (CAPRISA), Durban, South Africasijuisijali@gmail.com; 2 **Quarraisha Abdool Karim** is in the Centre for the AIDS Programme of Research in South Africa (CAPRISA), Durban, South Africaabdoolq2@ukzn.ac.za

**Keywords:** metabolites, mass spectrometry, enteric fever, typhoid, *Salmonella* Typhi, *Salmonella* Paratyphi A, human

## Abstract

The use of metabolomics could lead to improved diagnostics for enteric fever.

**Related research article** Näsström E, Nga TVT, Dongol S, Karkey A, Phat VV, Tuyen HT, Johansson A, Arjyal A, Thwaites G, Dolecek C, Basnyat B, Baker S, Antti H. 2014. *Salmonella* Typhi and *Salmonella* Paratyphi A elaborate distinct systemic metabolite signatures during enteric fever. *eLife* **3**:e03100. doi: 10.7554/eLife.03100

**Image** The mass spectrum of a plasma sample taken from a patient with enteric fever

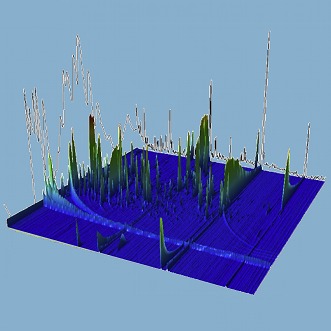

Enteric fever, also known as typhoid, is a disease that affects about 22 million people and causes about 200,000 deaths every year, according to conservative estimates (Buckle et al., 2012). Enteric fever is spread by bacteria belonging to the Salmonella genus, with two sub-species—*Salmonella* Typhi and *Salmonella* Paratyphi A—being responsible for most cases of the disease. And although the number of cases of enteric fever has fallen significantly over recent decades, there is a clear need for a diagnostic test for Salmonella that is rapid, affordable and accurate. Moreover, it is important to be able to distinguish between enteric fever caused by *Salmonella* Typhi and enteric fever caused by *Salmonella* Paratyphi A in order to ensure that the correct drugs are prescribed and to combat the development of antibiotic resistance.

The ideal diagnostic test offers high levels of sensitivity (that is, it is able to detect a high proportion of the people tested who have a particular disease), and also high levels of specificity (that is, it returns a ‘No’ for a high proportion of people without the disease). The current gold standard diagnostic tool for enteric fever is blood culture, but this has a low specificity and it is also challenging to perform consistently in regions with limited resources. As a consequence, misdiagnosis and mistreatment of enteric fever is common, which leads to poor clinical outcomes and the potential spread of antibiotic resistance. The development of nucleic acid and antigen-detection tests to diagnose enteric fever has faced similar challenges, with lower than expected sensitivity and specificity. One explanation for this could be that the levels of Salmonella in the blood tend to be very low at all stages of the disease (Darton et al., 2014).

Now, in *eLife*, Elin Näsström and co-workers at Umeå University, Oxford University (including clinical research units in Ho Chi Minh City and Kathmandu), and the London School of Hygiene and Tropical Medicine apply a promising new approach to this challenge (Näsström et al., 2014). Instead of trying to detect Salmonella in the blood during infection, they used a technique called metabolomics. The basic idea of this approach is that infection leads to metabolic changes, such that a person with enteric fever (or any infection) could have a profile of metabolites in their blood that is different to the metabolite profile of a healthy person. The challenge, therefore, is to identify a ‘metabolic fingerprint’ that can be used to detect enteric fever with high levels of sensitivity and specificity.

The application of metabolomics is relatively new in infectious diseases research compared to the application of genomics and proteomics. Despite this, screening the metabolome in blood plasma has identified useful prognostic profiles of several diseases, including sepsis (Langley et al., 2013). One of the major benefits of this technique is that it utilizes a pattern of biomarkers (that is, the various metabolites), as opposed to relying on just one host biomarker, as has been the focus of previous approaches.

Näsström et al. collected blood samples from 75 participants from South East Asia: 25 had enteric fever caused by *S.* Typhi*,* 25 had enteric fever caused by *S.* Paratyphi A, and 25 did not have enteric fever. Using a combination of gas chromatography and mass spectrometry, Näsström et al. identified 695 distinct peaks that were associated with different metabolites: from these they selected six peaks that had significantly different heights in the three groups of patients. This meant that they were able to tell if the patient had *S.* Typhi, *S.* Paratyphi A, or neither. That this mass spectrometric analysis was able to distinguish two Salmonella groups that share many similarities is remarkable. Moreover, in addition to its diagnostic potential, this new approach might also provide insights into the antigenic and physiological differences between the two strains.

There are, however, several issues that need to be addressed before this work will reach the clinic. The sample size in the study was relatively small (although it was randomly collected from a clinical trial repository). Näsström et al. also acknowledge that they did not perform any external validation, so it is not clear that the six biomarkers they identified will be relevant in other populations. It will be necessary to address these two points in future trials. Lastly, while negative controls were included, an expanded set of all-cause fever samples (that is, samples in which the enteric fever was caused by other organisms) will be needed to determine how well this panel of six biomarkers will perform in a real clinical setting.

Nevertheless, these findings represent an important step in the right direction. Näsström et al. acknowledge that mass spectrometers are too complex and expensive for routine use, but other more affordable technologies may be able to detect the six-metabolite fingerprint that they have identified. It might also be possible to use a similar approach to diagnose other bacterial infections.

Finally, given that antibiotic resistance is now a major public health challenge, and that we might be entering a ‘post-antibiotic era’ (Woolhouse and Farrar, 2014), these findings could represent a substantial technological advance and an important first step to addressing the problem of diagnosing and properly treating enteric fever and other blood-borne infections.
